# The Physical Activity Resource Assessment (PARA) instrument: Evaluating features, amenities and incivilities of physical activity resources in urban neighborhoods

**DOI:** 10.1186/1479-5868-2-13

**Published:** 2005-09-14

**Authors:** Rebecca E Lee, Katie M Booth, Jacqueline Y Reese-Smith, Gail Regan, Hugh H Howard

**Affiliations:** 1 Health and Human Performance, University of Houston, Garrison Gymnasium 104E, 4800 Calhoun Rd, Houston, TX 77204, USA; 2 Department of Psychology, University of Missouri-Kansas City, 4825 Troost, Suite 123, Kansas City, MO 64110, USA; 3 Psychology, University of Kansas, 315 Fraser Hall, 1415 Jayhawk Blvd., Lawrence, KS 66045-7556, USA; 4 Department of Psychology, Castleton State College,62 Alumni Dr., Castleton, VT 05735, USA; 5 Department of Geography, American River College, 4700 College Oak Dr., Sacramento, CA 95841, USA

## Abstract

**Background:**

Neighborhood environment factors may influence physical activity (PA). The purpose of this study was to develop and test a brief instrument to systematically document and describe the type, features, amenities, quality and incivilities of a variety of PA resources.

**Method:**

The one-page *Physical Activity Resource Assessment *(PARA) instrument was developed to assess all publicly available PA resources in thirteen urban lower income, high ethnic minority concentration neighborhoods that surrounded public housing developments (HDs) and four higher income, low ethnic minority concentration comparison neighborhoods. Neighborhoods had similar population density and connectivity. Trained field coders rated 97 PA resources (including parks, churches, schools, sports facilities, fitness centers, community centers, and trails) on location, type, cost, features, amenities, quality and incivilities. Assessments typically took about 10 minutes to complete.

**Results:**

HD neighborhoods had a mean of 4.9 PA resources (*n *= 73) with considerable variability in the type of resources available for each neighborhood. Comparison neighborhoods had a mean of 6 resources (*n *= 24). Most resources were accessible at no cost (82%). Resources in both types of neighborhoods typically had about 2 to 3 PA features and amenities, and the quality was usually mediocre to good in both types of neighborhoods. Incivilities at PA resources in HD neighborhoods were significantly more common than in comparison neighborhoods.

**Conclusion:**

Although PA resources were similar in number, features and amenities, the overall appearance of the resources in HD neighborhoods was much worse as indicated by substantially worse incivilities ratings in HD neighborhoods. The more comprehensive assessment, including features, amenities and incivilities, provided by the PARA may be important to distinguish between PA resources in lower and higher deprivation areas.

## Background

Regular physical activity has been associated with prevention of overweight and obesity, [1] as well as numerous other diseases [2]. Epidemiological studies have linked area of residence to physical activity [3-5]. Given consistently low rates of physical activity in the US, [2] it is important to develop an understanding of neighborhood factors that influence physical activity.

Residence in areas with high levels of material deprivation, an indicator of low area socioeconomic status (e.g., low median household income, and low educational attainment) has been associated with low levels of physical activity [3,5-7] as well as poor overall health [5,8] that may indicate low levels of physical activity. Despite the consistency of these findings, it is difficult to pinpoint the physical characteristics of material deprivation that contribute to physical inactivity. Deprived neighborhoods often have a lower proportion of owned homes compared to non-deprived neighborhoods, resulting in a low tax base for municipal improvements, have higher crime rates and reduced collective efficacy, [5,9,10] and have fewer goods and services available [7,11]. Although studies in two countries (UK, USA) have found that physical activity resources [12,13] vary by the socioeconomic status of the neighborhood, with lower SES areas having fewer physical activity resources, another study in Australia found greater access to physical activity resources in more deprived areas [14]. Although lack of access may be a driving factor to lower rates of physical activity in some deprived areas, there are likely additional qualitative elements of the physical activity resources that have not been well described or documented. For example, one study reported that attractiveness of public open space was associated with higher rates of walking [15]. However, there remain many elements of physical activity resources that have yet to be described or investigated in detail.

Although research in this area has been limited by access to geocoded databases to provide environmental information, the widespread adoption of Internet-based "user friendly" formats has resulted in a proliferation of research. Wider availability of these kinds of data have produced several studies investigating urban development constructs including walkability, a measure of how convenient and pleasant a neighborhood is for walking, [16,17] along with other street-scale elements, such as protected pedestrian pathways and availability of goods and services [18].

Despite these innovations, there remains no widely accepted system or protocol for describing or evaluating physical activity resources. The potential for endless permutations in type, equipment, size, shape, and condition of resources has hampered development of a common protocol. Further, until recently, little interest had been devoted to investigating the role that the physical environment plays in physical activity [19]. Recognition of physical activity as a universal good health recommendation, associated with the prevention of many chronic diseases, and success of policy and environmental approaches to manage health behaviors in other domains (e.g., tobacco control) has led researchers and funding agencies alike to pursue environmental approaches [19].

Available evidence suggests that there is a link between neighborhood factors and physical activity. Before this link can be clearly defined, it is important to develop strategies to assess neighborhood factors that may influence physical activity. The goals of this study were threefold. First, we aimed to develop an assessment protocol to describe physical activity resources based on existing literature and extensive pilot testing. Second, we assessed the type, quantity, features, amenities, and quality of all publicly accessible physical activity resources in urban neighborhoods available to public housing residents. Last, we compared the resources in the public housing neighborhoods to less deprived, comparison neighborhoods matched on age of housing and urban design (as measured by street network connectivity) and population density.

## Method

### Neighborhood Selection and Characteristics

Seventeen neighborhoods were selected for this study. Thirteen neighborhoods were defined as an 800 meter radius circumscribed around a public housing development managed by the state housing authority offices in Kansas City, Kansas and Missouri. Defining the neighborhood as the area within the boundaries of the circle has several advantages [20]. First, it captures all area to which a resident may be exposed on a daily basis during both foot and automobile travels. Second, the straight line distance allows for capture of distance traveled on footpaths and other "short cut" routes that may not be captured by using a street network strategy. Third, it may reduce the effect of spatial correlation that arises from using census boundaries where points near the boundary of the census area are influenced by factors in adjacent census areas, as housing developments were selected to be at least 1600 meters apart. Two housing developments violated this selection; however, they were separated by a major interstate that effectively eliminated daily exposure to the neighboring area.

Although Kansas City spans two states, it is a seamless city visually and practically. The public housing developments in this area serve a predominantly African-American population. Residents meet the 2003 US Department of Health and Human Service's Poverty guidelines, an annual household income of $18,400 or less per year for a family of four [21]. Aggregated neighborhood characteristics are presented in Table 1.

**Table 1:** Aggregated urban neighborhood characteristics.

Housing Development Neighborhoods	Median Household Income	Population Density	Percent Ethnic Minority	Street Connectivity
H01	29,299	5,548	62.1	118
H02	32,205	1,877	50.7	50
H03	16,902	3,925	71.1	108
H04	15,832	2,957	93.0	138
H05	11,930	1,770	98.1	94
H06	18,053	3,210	72.9	81
H07	18,644	2,960	70.3	54
H08	18,719	3,818	71.1	77
H09	22,519	4,087	60.6	93
H10	30,452	2,125	88.1	93
H11	34,303	2,779	71.4	84
H12	22,625	1,974	66.8	72
H13	25,844	3,218	52.6	98
Mean	22,871	3,096	71.4	89.2
(SD)	(7,005)	(1,073)	(14.3)	(24.2)

Comparison Neighborhoods				

C01	38,099	3,664	14.6	93
C02	48,383	2,889	9.1	99
C03	43,006	3,403	18.2	107
C04	39,970	2,167	10.7	105
Mean	42,364	3,031	13.2	101
(SD)	(4,493)	(660)	(4.1)	(6.3)

All housing development neighborhoods were located in urban areas that were predominantly lower income, with higher proportions of ethnic minorities. Four comparison neighborhoods were selected in areas that were similar in age and urban design, but higher in income with lower proportions of ethnic minorities. In comparison neighborhoods, an 800 meter radius was circumscribed around the centroid of an apartment complex or set of buildings with multiple family residences that were similar in size and appearance to public housing developments.

### Measures

#### Neighborhood level variables

United States Census data from the year 2000 were used to compute the aggregate median household income, population density and percentage of ethnic minorities for each neighborhood. All variables were drawn in aggregated form at the census block group level [22]. Neighborhoods often included parts of several block groups; thus, all values were calculated as weighted sums based on the overlap of housing development neighborhood buffer boundaries and block group boundaries [23,24]. For example, if a neighborhood buffer boundary encircled 30% of one block group with a median household income of $20,000, plus 20% of a second block group with a median household income of $25,000, plus 50% of a third block group with a median household income of $30,000, the weighted sum median income value for that neighborhood would be calculated as ($20,000 * 0.3) + ($25,000 * 0.2) + ($30,000 * 0.5) = $26,000. Population density was the number of people per square kilometer in each neighborhood. Proportion (reported as a percentage) of ethnic minorities was calculated as the sum of people identifying themselves as Black or African American alone; American Indian and Alaska Native alone; Asian alone; Native Hawaiian and Other Pacific Islander alone; some other race alone; or Hispanic, divided by the total population in that neighborhood. Street connectivity was calculated by counting the number of three or more street intersections in each neighborhood [18]. A three or more street intersection is any intersection where at least three streets are joined. These intersections may form a "T" or a "+" or a star shape when viewed from above.

#### Physical activity resources

The census of physical activity resources available to the general public was identified using a three step strategy [13]. First, Internet and telephone book searches were performed to generate an initial list of all physical activity resources in each neighborhood. Searches were done using an exhaustive list of terms identified previously [13] and built on by pilot testing for this study. Terms included physical activity, gym, fitness, dance studios, school, park, church, health clubs, bikeways, martial arts, sports, et cetera. All resources were mapped and verified initially by phone to confirm the presence and availability of the resource. Next, trained assessors conducted windshield surveys to confirm locations of resources and find any resources that had not been identified by existing databases.

Trained field coders assessed each physical activity resource on overall characteristics, the number, type and quality of features and amenities it possessed, and overall incivilities using the Physical Activity Resource Assessment (PARA) instrument (available from the principal investigator at ). Each physical activity resource was classified as a fitness club, a trail, a gym or sports facility, a park, a school, a church, or a community center. Resources that had multiple uses were coded based on the primary function of the resources. All resources were rated on hours of use, cost for use, and size (i.e., small, < ½ city block; medium, ½ city block < 1 city block; large, 1 city block or larger).

Data collectors counted and coded 25 unique possible elements of each physical activity resource that included 13 *features *used specifically for physical activity (e.g., basketball courts, soccer fields, playgrounds) and 12 *amenities *(e.g., benches, lighting, sidewalks). Each feature or amenity was also rated for quality by a three category quantitative system, which was developed based on extensive pilot testing of physical activity resources not in study neighborhoods. Ratings were listed as 3 "good," 2 "mediocre," and 1 "poor," with specific operational definitions developed by the research team for each item in each category. Definitions were constructed based on objective standards of quality. For example, an outdoor soccer field's rating of good was defined as "Field has uniform grass coverage and is well-mowed, no trash or debris on field; nets, if furnished are intact;" mediocre was defined as "Grass coverage may be sparse in a few places, grass may be too high, some trash or debris on field;" and poor was defined as "Grass coverage may be poor in 50% or > of the field, rough surface, hazards and/or trash on the field." Good sidewalks were defined as "Sidewalk is smooth, clear of debris," while mediocre sidewalks had "some debris, cracks or uneven surfaces, but [were] otherwise usable," and poor sidewalks had "major damage and need repair, almost unusable."

Each physical activity resource was also rated on overall incivilities. Incivilities included 9 elements that would reduce the pleasure associated with using that physical activity resource. These included auditory annoyances, broken glass, dog refuse, unattended dogs, evidence of alcohol and substance use, graffiti, litter, not enough grass or overgrown grass, sex paraphernalia, and vandalism [25]. *Incivilities *is a term that was originally coined by criminologists Wilson and Kelling [26] and has been investigated in sociological and anthropological contexts to describe the quality and social order of a neighborhood [27,28]. The presence of incivilities has been associated with less physical activity [25] and other poor health outcomes [10]. Incivilities were coded on a four category rating system of 4 "not present," 3 "a little," 2 "a medium amount," and 1 "a lot." Objective operational definitions were created and pilot tested for each item. For example, unattended dogs were defined as, good "1 dog unattended," mediocre "2–4 dogs unattended; may be associated noise," and poor "5 or > dogs unattended, definitely unsafe, may be associated noise." Litter was defined as, good "A few items (< 5) are on the ground," mediocre "Several items (5–10) are on the ground," and poor "Many items are on the ground (11+)."

### Procedures

The physical activity resource assessment instrument was developed over a nine month period. The instrument was pilot tested and revised numerous times to achieve the final form. Reliability tests of a 10% overlap showed good reliability (*r*s > .77).

After neighborhoods were selected, the physical activity resource census was developed using the above described method. Trained field assessors (three doctoral candidates in psychology) used the instrument to systematically describe each physical activity resource. All assessments were conducted during daylight hours in the spring, summer and fall seasons when the ground was free from snow or ice. Assessors were accompanied by a second student for safety reasons in the housing development neighborhoods, and procedures included safety protocols in case of imminent perceived danger. Field assessments typically took about 10 minutes to complete; however, in a few cases of larger resources (e.g., a large park) the instrument could take up to 30 minutes to complete. Data were entered and proofed by trained graduate assistants. All analyses were conducted using SPSS [29].

## Results

### Neighborhood characteristics

Aggregate neighborhood characteristics are described in Table 1. Housing development neighborhoods had a median household income range of $11,930–$34,303, (*M *= $22,871, *SD *= $7,004), a population density range of 881–2,761, (*M *= 1,541, *SD *= 534), a non-white population range of 50.7%–98.1% (*M *= 71.4%, *SD *= 14.3%), and a street intersections range of 50–138 (*M *= 89.2, *SD *= 24.2). Comparison neighborhoods had a median household income range of $38,099–$48,383, (*M *= $42,364, *SD *= $4,493), a population density range of 1,079–1,824, (*M *= 1,508, *SD *= 328), an ethnic minority population range of 9.1%–18.2% (*M *= 13.2%, *SD *= 4.1%), and a street intersections range of 93–107 (*M *= 101, *SD *= 6.3).

**Figure 1:**
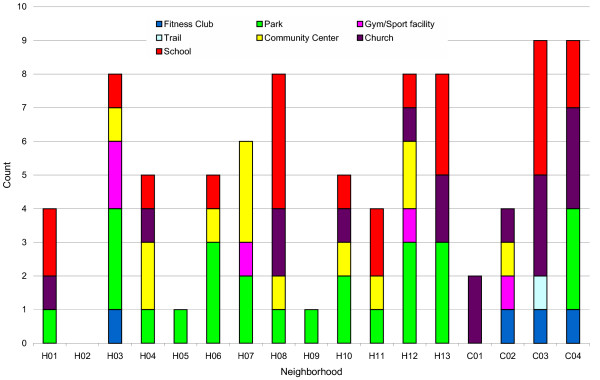
Physical Activity Resources by Neighborhood

Housing development neighborhoods had a range of 0 to 8 physical activity resources (*M *= 4.85, *SD *= 2.82), including fitness clubs, parks, sport facilities, community centers, churches, and schools, with considerable variability in the type of resources available for each neighborhood. As shown in Figure 1, one in three PA resources in HD neighborhoods were parks (*n *= 22, 35%), possibly reflecting preferences of early city developers. One fourth of PA resources were public school yards (*n *= 16, 25%) illustrating an important, and underrecognized, role that public schools play in communities. Most (*n *= 8, 62%) communities also had access to a community center. Comparison neighborhoods had a range of 2 to 9 PA resources (*M *= 6, *SD *= 3.56), including fitness clubs, parks, sport facilities, trails, community centers, churches, and schools. As shown in Figure 1, 38% (*n *= 9) of the resources in comparison neighborhoods were churches, but only one neighborhood (25%) had access to a community center. Most resources were freely accessible at no cost (82%), and appeared evenly distributed throughout neighborhoods.

Table 2 presents the physical activity features and resource amenities and their quality by neighborhood. Thirteen possible physical activity features within each resource were assessed for availability and quality. HD neighborhoods had slightly more physical activity features within each resource (*M *= 2.71, SD = 1.65) than did resources in comparison neighborhoods (*M *= 2.17, SD = 1.63). Although not shown in the tables, it is interesting to note that in HD neighborhoods, fitness clubs and community centers had the most physical activity features available, on average, (M = 4, SD = 1.91); however, in comparison neighborhoods, parks had the most physical activity features available (M = 4.67, SD = 1.53). Sport facilities had the lowest average number of physical activity features within each resource available for both the HD neighborhoods (M = 1.25, SD = .50), and the comparison neighborhoods (which had none of the features on the assessment form). In HD neighborhoods, quality ratings for physical activity features within resources ranged from 1 to 3 (M = 2.45, SD = .81), and from 1 to 3 (M= 2.41, SD = .96) at comparison neighborhoods.

**Table 2:** Mean count and quality ratings for amenities and physical activity features by neighborhood type.

HD ID	Count of Resources	Mean Count of Features	Mean Quality of Features	Mean Count of Amenities	Mean Quality of Amenities
H01	4	1.25	1.25	1.75	2.06
H02	0	0	0	0	0
H03	8	2.25	2.64	4.37	2.60
H04	5	4.20	2.25	4.40	2.54
H05	1	2.00	3.00	4.00	2.25
H06	5	2.60	2.45	2.80	2.68
H07	6	2.33	2.32	4.83	2.47
H08	8	2.63	2.50	3.50	2.27
H09	1	2.00	3.00	5.00	2.20
H10	5	4.00	2.89	4.40	2.31
H11	4	3.25	2.80	3.25	2.28
H12	8	3.00	2.60	3.88	2.36
H13	8	2.25	2.31	3.63	2.38

Total	63				
*Mean*		2.71	2.45	3.79	2.40
*(SD)*		(1.65)	(.81)	(2.16)	(.68)

C01	2	3.00	2.75	2.00	2.00
C02	4	2.00	2.17	4.50	2.69
C03	9	1.22	1.89	1.00	1.72
C04	9	3.00	2.96	4.44	2.81
Total	24				
*Mean*		2.17	2.41	2.96	2.32
*(SD)*		(1.63)	(.96)	(2.42)	(.97)

Twelve possible amenities were assessed for availability and quality at each resource. HD neighborhoods had more amenities per resource, on average (*M *= 3.79, SD = 2.16) than did comparison neighborhoods (M = 2.96. SD = 2.42). Although not presented in the table, on average, community centers had the most amenities available for both the HD neighborhoods (M = 5.5, SD = 1.98) and the comparison neighborhoods (N = 8, SD = 0). In HD neighborhoods, churches had the fewest amenities available (M = 1.50, SD = 1.31), although in comparison neighborhoods, fitness clubs had the fewest amenities available (M = 1.67, SD = 1.53). For amenities, quality ratings ranged from 1 to 3 (M = 2.40, SD = .68) in HD neighborhoods, and from 2 to 3 (*M *= 2.32, SD = .97) in comparison neighborhoods. However, quality ratings within each neighborhood varied widely.

**Figure 2:**
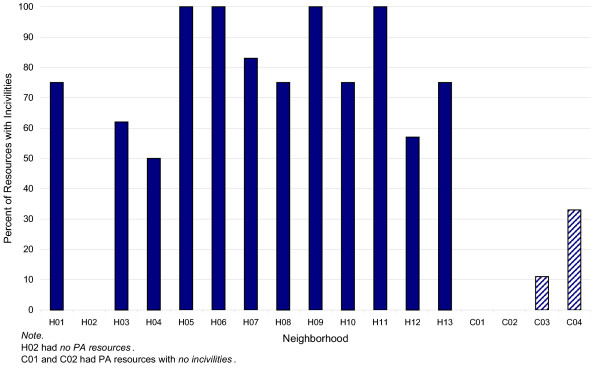
Percent of PA Resources with Incivilities by Neighborhood

Eighty percent of resources in all HD neighborhoods had incivilities (*M *= 1.81 per resource, SD = 1.72). In contrast, incivilities were found at only 11% of the PA resources in only half of the comparison neighborhoods (*M *= .29, SD = .75). This relationship was significant (*t *= 12.60, *p *< .001) as illustrated in Figure 2. Litter was the most frequently reported incivility for HD resources (65%, *N *= 41), with 20 resources having litter ratings of a medium amount to a lot. Broken glass was found at 25% of the resources (*N *= 16), with 14 ratings of a medium amount to a lot. Twelve resources (19%) had evidence of alcohol use, with 7 ratings of a medium amount to a lot. An auditory annoyance was reported at 16% (*N *= 10) of HD resources, with 8 of the resources having auditory annoyance ratings of a medium amount to a lot, mostly for traffic noise. Graffiti or tagging was found at 14% (*N *= 9) of the resources, with 6 having a medium amount to a lot. Nine of the resources (14%) lacked grass, while 10 (16%) of the resources had overgrown grass. Dog refuse (*N *= 1), unattended dogs (*N *= 2), evidence of substance use (*N *= 1), and vandalism (*N *= 1) were seen at less than two percent of the resources, and sex paraphernalia was not present at any. Comparison neighborhoods had few incivilities. Litter was present at 17% (*N *= 4) of the resources, with 2 having a medium amount to a lot. Graffiti, overgrown grass, and vandalism were all found at one (4%) of the resources, with ratings of a little bit to a medium amount.

## Discussion

This study (1) developed the PARA instrument that provides a brief, reliable and effective strategy to objectively assess neighborhood factors that may influence physical activity by describing the type, quantity, features, amenities and incivilities of physical activity resources, and (2) used the PARA to evaluate the physical activity resources in thirteen lower income, high ethnic concentration neighborhoods that surrounded public housing developments in comparison to physical activity resources in higher income, low ethnic minority concentration neighborhoods.

Lower income, higher ethnic concentration, housing development neighborhoods varied widely in the number and type of resources that were available for physical activity. All but one of the neighborhoods had access to parks, and most had accessible public school yards and community centers. The high number of parks found in housing development neighborhoods likely reflects city planners and landscape architects from over a century ago, such as Frederick Law Olmstead [30] who advocated for park development for the civilizing influence of "neighborly recreation" and its benefits to human health. As was posited then, more recent data suggest that people who live near attractive, public open spaces may be almost twice more likely to walk at moderately active levels than were those who do not have access to public open spaces [31]. Higher income, low ethnic concentration, comparison neighborhoods also varied widely in the number and type of physical activity resources available. The neighborhoods were selected to be similar in age and urban design; thus, the housing development neighborhoods and comparison neighborhoods might have similar amounts of parks, schoolyards and other public structures resulting from similar urban planning strategies. Although efforts were made to select appropriate comparison neighborhoods for the purposes of the current study, these neighborhoods do not represent the entire universe of possible neighborhoods.

Although the net number of physical activity resources did not vary by neighborhood income and ethnic concentration, the overall environment of physical activity resources was strikingly different in the neighborhoods, and suggests that evaluating merely the presence or absence of physical activity resources may be an overly simplistic way to investigate access to resources. In this sample, incivilities were consistently present and conspicuously bad and offensive at physical activity resources in lower income, higher ethnic concentration neighborhoods. In comparison, we found no incivilities at the resources in half of the comparison neighborhoods, and very few incivilities at the resources of the other half. It would seem to make sense that the presence of litter and debris, as well as lack of apparent maintenance might be important detractors from using a physical activity resource for physical activity, because people who walk more frequently typically rate their environment more positively [31]. In fact, a high proportion of incivilities might suggest lack of attention to an area, and might even encourage less desirable behavior (e.g., drug trafficking, prostitution) clearly not promoting favorable conditions for recreational physical activity [25].

Perhaps most distressing about these findings is the suggestion that the enduring relationship between social inequalities and poor health in an ostensibly wealth country, the US, is complex and appalling. The results suggest that merely building a park in a deprived area may be insufficient for insuring its intended use and maintenance. It is critical to provide ongoing support for maintenance and civic improvements. There is a deep need for policy makers and political leaders to work with communities to improve the quality of publicly available physical activity resources to improve the quality of life for all.

## Conclusion

Previous work suggests that convenient neighborhood resources are critical for meeting physical activity recommendations [31,32]. An important contribution of this study is the inclusion of quality and incivility ratings in our instrument. To date, investigations that have examined physical activity resources have been limited to investigating the type and accessibility of resources [13,25]. This study is among the first to comprehensively evaluate a broad range of physical activity resources on a number of dimensions. At the same time, the PARA instrument was developed to be easily and rapidly administered, promoting broad scale dissemination and relatively, easily translatable outcomes for policy implementation. Future research is needed that tests the associations among these physical activity resource dimensions and individual physical activity levels to guide individual and environmental intervention development.

## Authors' contributions

REL conceived of the study, and directed all aspects of the study including development, assessment and analyses, and lead the writing of the manuscript. KMB, JRS, GR all helped to develop measures, conduct assessment and analyses and assisted with the writing of the manuscript. HH provided geographic support including neighborhood maps, location of resources and assistance with variable construction, as well as assisting with the writing of the manuscript. All authors read and approved the final manuscript.
